# Lomitapide ameliorates middle cerebral artery occlusion‐induced cerebral ischemia/reperfusion injury by promoting neuronal autophagy and inhibiting microglial migration

**DOI:** 10.1111/cns.13961

**Published:** 2022-09-02

**Authors:** Yangmin Zheng, Yue Hu, Ziping Han, Feng Yan, Sijia Zhang, Zhenhong Yang, Fangfang Zhao, Lingzhi Li, Junfen Fan, Rongliang Wang, Yumin Luo

**Affiliations:** ^1^ Institute of Cerebrovascular Disease Research and Department of Neurology Xuanwu Hospital of Capital Medical University Beijing China; ^2^ Beijing Geriatric Medical Research Center and Beijing Key Laboratory of Translational Medicine for Cerebrovascular Diseases Beijing China

**Keywords:** autophagy flux, drug repurposing, ischemic stroke, lomitapide, microglia migration

## Abstract

**Aims:**

Stroke has a high incidence and is a disabling condition that can lead to severe cognitive, motor, and sensory dysfunction. In this study, we employed a drug repurposing strategy to investigate the neuroprotective effect of lomitapide on focal ischemic brain injury and explore its potential mechanism of action.

**Methods:**

Experimental cerebral ischemia was induced by middle cerebral artery occlusion (MCAO) in adult male C57BL/6 mice and simulated by oxygen–glucose deprivation in N2a‐BV2 cells in co‐cultivation.

**Results:**

Lomitapide significantly increased the survival rate, reduced the neuronal tissue loss, and improved the neurological function after MCAO. Furthermore, lomitapide could increase the expression of LC3‐II, reduce the expression of P62 and LAMP2, promote autophagic flux, and inhibit apoptosis by increasing and inhibiting the expression of the apoptosis‐associated proteins Bcl‐2 and Bax, respectively. In addition, lomitapide inhibited the migration of pro‐inflammatory microglia.

**Conclusion:**

Lomitapide is a lipid‐lowering drug, and this is the first study to explore its protective effect on ischemic nerve injury in vitro and in vivo. Our results suggest that lomitapide can be repositioned as a potential therapeutic drug for the treatment of stroke.

## Graphical abstract

Lomitapide ameliorates cerebral ischemia/reperfusion injury via promoting neuron autophagy and inhibiting microglia migration and can be repositioned as a potential therapeutic drug for stroke treatment.

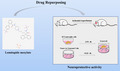

## 1 INTRODUCTION

Ischemic stroke is a major disease that endangers health and life of the elderly. It is characterized by high incidence, mortality, and disability rates. During acute cerebral infarction, cerebral blood supply is insufficient wherein ischemia and hypoxia lead to brain cell metabolic disorder and even nerve cell death, resulting in irreversible damage to the tissue. At present, there are no specific drugs for the treatment of stroke, and the accepted post‐stroke measures are early improvement of cerebral blood perfusion and neuroprotective treatment. However, the limited time window for thrombolytic therapy limits its clinical application, making it particularly important to develop new and effective drugs, and explore novel targets for the treatment of ischemic stroke. Therefore, extensive study of the mechanism of cerebral ischemia and developing new drugs based on specific drug targets are critical.

Research and development of new drugs involves “large investment and small output.” Even after the successful launch of new drugs, some drugs will be forced to be withdrawn from the market due to adverse reactions. In order to shorten the development cycle of new drugs, reduce risks, and improve the success rate of new drug development, “drug repurposing” has attracted considerable attention. Drug repurposing is based on previous research and development wherein detailed drug formula, mechanism of action, and safety information are known. Therefore, compared to developing new drugs, drug repurposing has obvious advantages of low research cost, low risk, high success rate, and faster clinical trials.^1^, ^2^ Lomitapide mesylate is an inhibitor of microsomal triglyceride transfer protein (MTP) used for the treatment of homozygous familial hypercholesterolemia. It can directly bind to MTP and inhibit the assembly of apoB‐lipoprotein in the liver and intestinal epithelial cells, inhibit chylomicron and very low‐density lipoprotein production, and reduce the concentration of low‐density lipoprotein in plasma.^3^ Our previous computer model screening indicated lomitapide mesylate as an inhibitor of histone deacetylase (HDAC). Broad‐spectrum inhibitors of HDACs have been demonstrated to regulate Treg function and the phenotypic shift of macrophage/microglia in ischemic stroke.^4^ We also found that inhibition of HDAC was protective against ischemic brain injury.^5^, ^6^, ^7^ Therefore, we initiated this study to verify the neuroprotective effect of lomitapide and explore its possible mechanism of action.

## 2 METHODS

### 2.1 Cell culture

For primary culture of cortical neurons, cerebral cortical neurons were collected from 18‐day‐old C57BL/6J mice. The brain tissue of fetal mice was obtained by micromanipulation; the meninges and microvessels were removed; and the cortex was carefully separated, cut, and digested with 0.05% trypsin at 37°C for 5 min. The extracted cells were inoculated onto poly‐L‐lysine‐coated (Sigma) cell culture plates in Dulbecco's modified Eagle medium (DMEM, Gibco) containing 10% fetal bovine serum (FBS, Gibco), with an inoculation density of approximately 1.0 × 10^6^ cells/ml. The next day, the cells were cultured in Neurobasal medium containing 2% B27 (Gibco) and 1% penicillin/streptomycin, and the solution was changed every 3 days with incubation under 37.5% CO_2_ + 95% O_2_.

Murine BV2 and Neuro‐2a cells (China Infrastructure of Cell Line Resources) cells were cultured in DMEM supplemented with 10% FBS and 1% penicillin/streptomycin at 37°C with 5% CO_2_. BV2 and Neuro‐2a were adherent cells and were subcultured every 2 to 3 days.

To initiate oxygen–glucose deprivation (OGD), primary neuronal cells were cultured in an ischemia‐mimetic solution in a hypoxia incubator chamber filled with 95% N_2_/5% CO_2_ at 37°C for 2.5 h, whereas Neuro‐2a cells were cultured in the same conditions for 4 h. After OGD treatment, lomitapide (Selleck Chemicals) in gradient concentration (0.01, 0.1, and 1 μM) was administered for 24 h. Lomitapide was prepared in a 5 mM stock solution with DMSO.

For co‐culture studies, BV2 microglia and Neuro‐2a cells were co‐cultured using transwell cell culture inserts (Dow Corning). Before co‐culture, BV2 microglia were treated with LPS, while Neuro‐2a cells were treated with OGD. Neuro‐2a cells were grown in the plate, and BV2 cells were seeded on the microporous membrane of the transwell cell culture inserts.

### 2.2 Cell viability assay

Cell viability assay was performed using the CellTiter‐Glo® Luminescent Cell Viability Assay (Promega) according to the manufacturer's recommended protocol.

### 2.3 Mouse model of middle cerebral artery occlusion (MCAO)

All animal experiments were approved by the Institutional Animal Care and Use Committee of Xuanwu Hospital of Capital Medical University and were carried out in accordance with the principles outlined in the National Institutes of Health's Guide for the Care and Use of Laboratory Animals.

Male C57BL/6J mice (2‐month‐old) weighing 22–25 g were purchased from Vital River Laboratory Animal Technology Co. Ltd. MCAO model was induced using the intraluminal filament method as previously described.^8^ The middle cerebral artery of mice was obstructed for 45 min, and the embolus was pulled out to restore the blood flow. Body temperature was maintained at 37.0 ± 0.5°C during the procedure. After the operation, the animals were raised in standard environment. Thirty‐six mice were randomly divided into three groups: sham (*N* = 12), ischemia (MCAO 45 min)/reperfusion (I/R) + vehicle (*N* = 12; I/R + Veh), I/R + Lomitapide (0.5 mg/kg, po q.d) treatment for 14 days (*N* = 12; I/R + Lomitapide). Lomitapide was dissolved in saline to a concentration of 0.25 mg/ml, and mice were administered 250 μl of lomitapide intragastrically immediately after reperfusion for 14 consecutive days. The experimental procedures and pharmacological manipulations are depicted in the flowchart in Figure 2A.

### 2.4 Neurological function scoring

For adhesive removal testing,^9^ tape strips (30 × 40 mm) were stuck on the palm of the mouse's front paw, and the time point (Contact time) when the mouse's mouth first contacted the tape and the time point (Remove time) when the tape was torn off were recorded. If the tape was not torn off after 120 s, it was recorded as 120 s. For the beam walking test,^10^ the mouse was placed on one end of the balance beam (length = 120 cm, width = 2 cm, height = 30 cm) and then instinctively went to the other end; the time of empty/sliding of the animal's hind limbs within 1 m was recorded. Each test was repeated three times with a suspension time of 2 min.

### 2.5 Neuronal tissue loss

All mice were sacrificed under anesthesia 14 days after MCAO, brain slices (25 μm thick) were collected at 0.3 mm intervals, and the first slice in each interval was stained with NeuN antibody (MAB377, Millipore).^11^ Volume of neuronal tissue loss was calculated using ImageJ software (National Institutes of Health). Infarct volume was normalized to the contralateral hemisphere. The percent of neuronal tissue loss was expressed using the following equation: area of the contralateral hemisphere—non‐infarcted area of the ipsilateral hemisphere/area of the contralateral hemisphere × 100%.

### 2.6 Western blotting

Proteins extracted from ipsilateral brain tissue of mouse and cell lysates with radioimmunoprecipitation assay (RIPA) lysis buffer (1% Triton X‐100, 50 mmol/L Tris–HCl, pH 7.5, 100 mmol/L NaCl) containing protease inhibitors and phosphatase inhibitors (Sigma cocktail, Sigma‐Aldrich, St. Louis, MO, USA) were separated using 12% sodium dodecyl sulfate polyacrylamide gel electrophoresis and transferred onto a nitrocellulose filter. Membranes were blocked for 1 h in 5% dried skimmed milk and incubated with the following primary antibodies overnight at 4°C: anti‐P62 (PM045, MBL, Japan), anti‐LAMP2 (sc‐18822), anti‐LC3B (M186‐3, MBL), anti‐Bax (2772, Cell Signaling Technology), anti‐Bcl‐2 (2870, Cell Signaling Technology), and anti‐GAPDH (sc‐3650062). The blots were incubated with horseradish peroxidase‐conjugated secondary antibodies (Santa Cruz Biotechnology) for 1 h and visualized using an enhanced luminescence kit (Millipore).

### 2.7 Co‐immunoprecipitation

For co‐immunoprecipitation (co‐IP), cell lysates from N2a cells were centrifuged at 13,000 × *g* for 10 min at 4°C, and the supernatants were pre‐cleared with 20 μl protein A/G agarose beads for 2 h and separately incubated overnight with either anti‐LAMP2 (sc‐18822) or anti‐P62 (PM045, MBL) at 4°C, followed by 2 h incubation with protein A/G plus agarose (Santa Cruz Biotechnology), also at 4°C. After washing 3 times with the IP buffer, the immunoprecipitated complexes were eluted by boiling with SDS loading buffer for 6 min. The following immunoblot analyses were performed with the antibodies: anti‐P62 (PM045, MBL), anti‐LAMP2 (sc‐18822), and anti‐LC3B (M186‐3, MBL).

### 2.8 Immunofluorescence staining

Mouse brain tissue sections and cells were incubated with following specific antibodies: anti‐LC3B (PM036, MBL), anti‐Iba‐1 (019‐19741, Wako), anti‐NeuN (MAB377, Millipore), anti‐P62 (ab56416, Abcam), anti‐LAMP2 (sc‐18822), anti‐ki67 (ab15580, Abcam), anti‐MAP2 (ab11267, Abcam), anti‐MBP (ab62631, Abcam), and anti‐Iba1 (019‐19741, Wako Pure Chemical Industries, OSA) in a humidified container for 12 h at 4°C, followed by incubation with fluorescent conjugated secondary antibodies (Santa Cruz Biotechnology).

### 2.9 Terminal transferase dUTP nick end labeling (TUNEL) assay

Terminal transferase dUTP nick end labeling staining was performed to evaluate apoptosis of neurons by using In Situ Cell Death Detection kit according to the manufacturer's protocol (Roche Applied Science).

### 2.10 Transwell migration assay

Migration of BV2 cells was examined using transwell inserts fitted with polycarbonate filter (8‐μm available pore size, #3422; Corning Inc., NY, USA). Neuro‐2a cells in 10% FBS‐containing medium were grown in a plate, and BV2 cells in FBS‐free medium were seeded on microporous membrane of transwell cell culture inserts. Following 24 h incubation with lomitapide, the cells in the upper compartment were fixed with 4% paraformaldehyde solution for 20 min and stained with crystal violet solution for 10 min. The cells in the upper chamber were removed with a cotton swab, and the chamber was examined under a microscope. Subsequently, crystal violet was dissolved with 33% acetic acid, and the absorbance was measured at 450 nm using a microplate reader.^12^

### 2.11 Statistical analysis

All data were expressed as mean ± SEM. Statistical analyses were performed using GraphPad Prism 7.0 (GraphPad Software Inc.). The D'Agostino and Pearson omnibus normality test was used to analyze the normality of data, and the data were found to be normally distributed. Comparison between the two groups was tested using the *t*‐test. Comparison between multiple groups was performed using one‐way ANOVA or two‐way ANOVA, with Tukey's post hoc test, and results with *p*‐values <0.05 were considered significant.

## 3 RESULTS

### 3.1 Lomitapide ameliorates OGD‐induced cell injury

**FIGURE 1:**
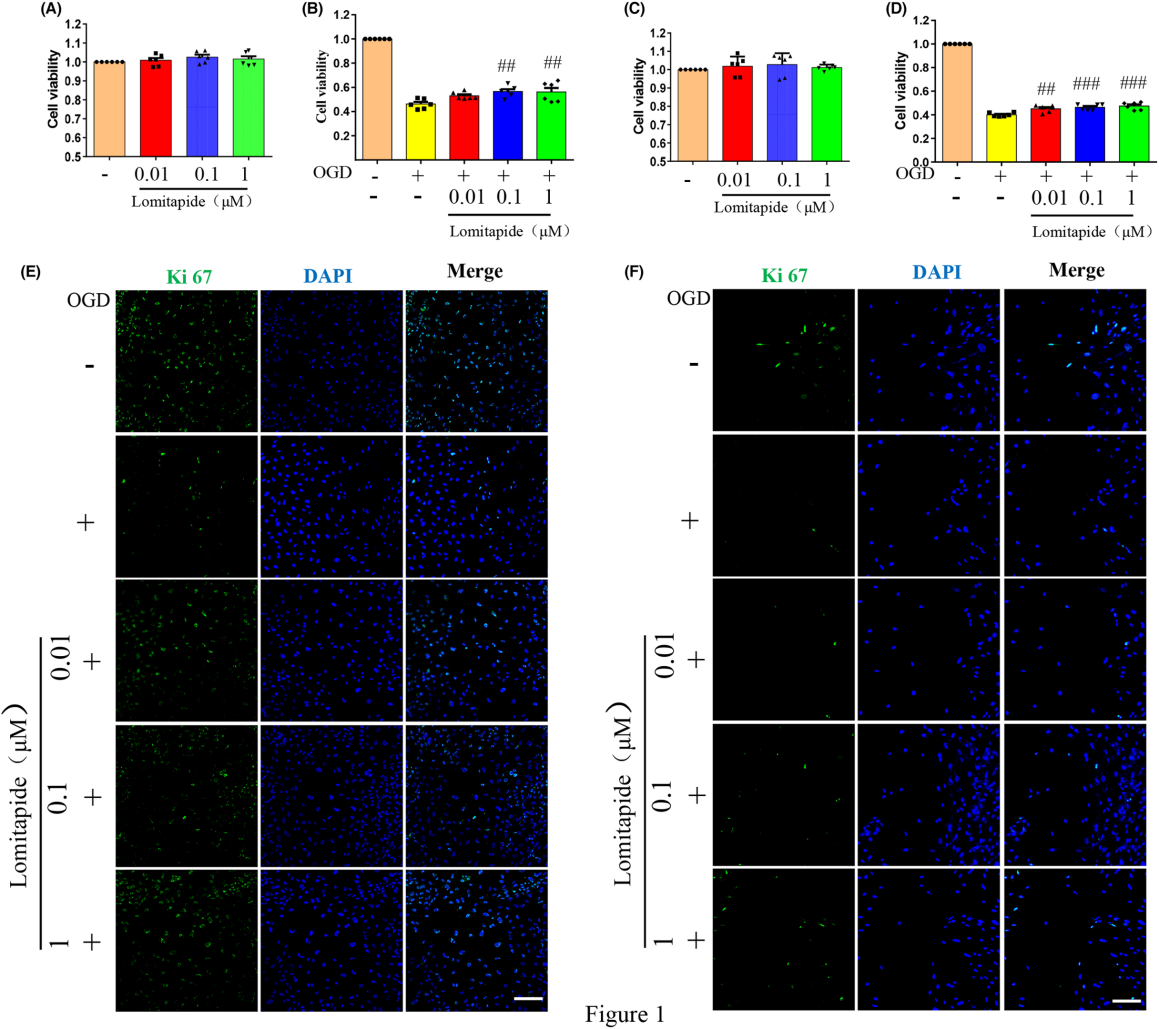
Lomitapide ameliorates OGD‐induced cell injury in primary neurons and Neuro‐2a cells. Lomitapide had no cytotoxic effect (A) and could alleviate cell damage induced by OGD (B) in Neuro‐2a cells. Lomitapide had no cytotoxic effect (C) and could alleviate cell damage induced by OGD (D) in primary neurons. *n* = 6, ^#^Compared with the OGD group, the level of statistical significance is indicated as ^#^ *p* < 0.05, ^##^ *p* < 0.01, and ^###^ *p* < 0.001, one‐way ANOVA followed by Tukey's multiple comparison test. Representative immunofluorescence images of ki67 (green) in Neuro‐2a cells (E) and primary neurons (F). The bars indicate 100 μm

In cell stability tests, we found that lomitapide had no toxic effect on mouse primary neurons and Neuro‐2a cell lines, compared with the control group at gradient concentrations (0.01, 0.1, and 1 μM) (Figure 1A,C). For the cell survival experiments, the OGD cell model was used to simulate ischemic stroke. After lomitapide treatment for 24 h and OGD for 2.5 or 4 h, we found that lomitapide could alleviate cell damage caused by OGD and improve cell viability in a concentration‐dependent manner compared with the OGD model group, in mouse primary neuron cells and Neuro‐2a cell lines, and the difference was statistically significant (Figure 1B,D, *p* < 0.0001). Immunofluorescence staining of ki67 showed that lomitapide reversed the decrease in cell activity induced by OGD in mouse primary neurons and Neuro‐2a cell lines (Figure 1E,F). These results suggest that lomitapide has a potential role in protecting neurons from ischemia and hypoxia, which was further verified in animal models.

### 3.2 Lomitapide improves recovery of neurological function and reduces neuronal tissue loss in ischemic mice

**FIGURE 2:**
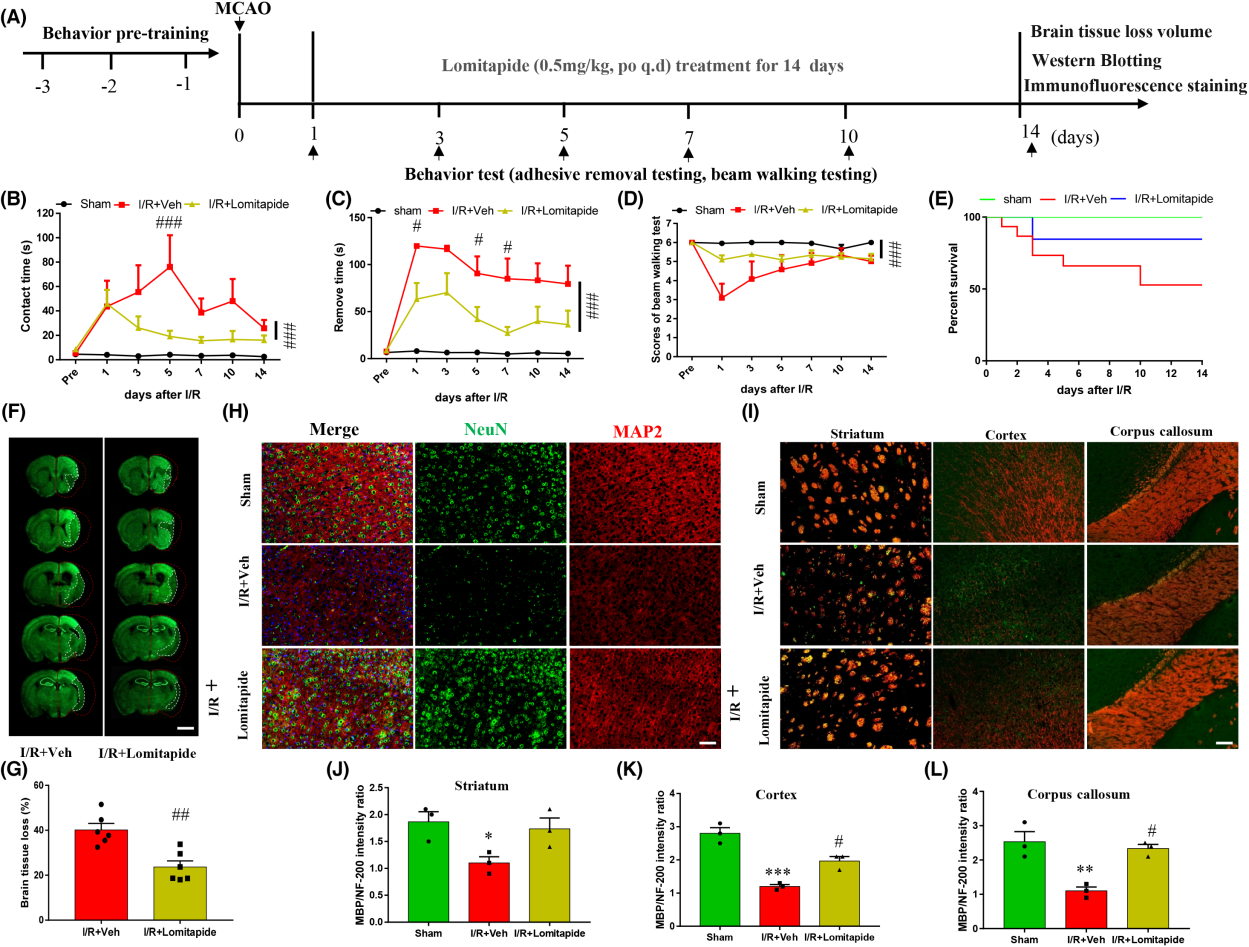
Lomitapide promotes recovery from neurological deficits and decreases neuronal tissue loss in ischemic mice. A representative flowchart of the experimental procedure in this study (A). Sensorimotor functions were evaluated using the adhesive removal test; the latencies to contact (B) and remove (C) the tape were recorded. Motor coordination and integration functions were evaluated using the balance beam test (D). (*n* = 6–12, ^#^ *p* ≤ 0.05, ^###^ *p* ≤ 0.001, I/R + Lomitapide vs. I/R + Veh group, respectively, two‐way ANOVA followed by Tukey's multiple comparison test). The 14‐day survival rates of each group after MCAO (E), *n* = 12. Representative images and quantification of neuronal tissue loss in different groups at 14 days after I/R (F, G), *n* = 6, ^##^ *p* ≤ 0.01, I/R + Lomitapide vs. I/R + Veh group, unpaired *t*‐test, the bars indicate 2 mm. Representative immunofluorescence images of co‐localization of MAP2 (red) and NeuN (green) in the sham, I/R + Veh, and I/R + Lomitapide groups. DAPI (blue) indicates cell nuclei (H). Representative immunofluorescence images of MBP (red) and NF‐200 (green) in the coetex, corpus callosum, and striatum among the three groups (I) The bars indicate 50 μm. Quantification of the relative ratio of MBP to NF‐200 immunostaining intensity in the striatum (J), coetex (K), and corpus callosum (L), *n* = 3, *p ≤ 0.05, **p ≤ 0.01, and ***p ≤ 0.001 vs. sham group; ^#^ *p* ≤ 0.05 vs. I/R + Veh group

To further evaluate the neuroprotective effect of lomitapide, we used a MCAO mouse model to validate this result. Mice were trained for neurological functional tests 3 days before MCAO and were administered lomitapide 0.5 mg/kg daily for 14 days after MCAO.^13^, ^14^ Neurological function was scored 1, 3, 5, 7, 10, and 14 days after MCAO, as shown in Figure 2A. We evaluated the sensorimotor ability of mice by using the adhesive removal test. As shown in Figure 2B,C, mice in the lomitapide treatment group spent significantly shorter time from contacting (vs. I/R + Veh group, *p* = 0.0018) the tape to removing (vs. I/R + Veh group, *p* < 0.0001) the tape off from the left front paw compared with the model group, showing better sensorimotor ability. A balance beam test was used to evaluate the motor coordination and integration function of mice. We found that lomitapide significantly improved the motor function of MCAO mice (vs. I/R + Veh group, *p* = 0.0002) and was statistically significant compared with the control group (Figure 2D). Moreover, we observed the survival of mice in the sham, I/R, and I/R + Lomitapide groups after ischemic stroke and plotted the 14‐day survival curve of cerebral ischemia–reperfusion in each group. The results showed that lomitapide treatment significantly improved the survival rate of mice 14 days after MCAO compared with the I/R group (Figure 2E). We calculated the percentage of brain tissue loss using neuronal nuclei (NeuN) immunostaining (Figure 2F,G). The results showed that the model group mice had obvious neuronal tissue loss and that lomitapide treatment could significantly reduce the degree of neuronal tissue loss (40.19 ± 2.816 vs. 23.69 ± 2.707, *p* = 0.0018). In addition, double immunofluorescence staining for the neuronal markers MAP2 and NeuN (Figure 2H) revealed loose and disordered cell arrangement and decreased number of neurons in the I/R group compared with the sham group, which was reversed by lomitapide treatment. MBP is a specific protein expressed in mature myelin sheath, which will be lost in demyelinating lesions; NF200 is a marker protein of nerve filament, which can be exposed after myelin sheath injury. The I/R group showed loose and even broken morphology of MBP and severely damaged demyelination in the cortex, corpus callosum, and striatum region, compared with the sham group, the exposure of NF‐200 was increased. In contrast, in the lomitapide treatment group, the MBP tended to be morphologically intact and NF‐200 levels decreased. (Figure 2I). Additionally, MBP/NF200 can indicate the degree of white matter damage. Statistical analysis of MBP/NF200 fluorescence intensity ratio (Figure 2J–L) suggested that I/R caused a decrease in this ratio, while lomitapide significantly increased the value after treatment in the cortex and corpus callosum region (*p* < 0.05), confirming that lomitapide treatment could promote the repair of myelin protein in white matter surrounding infarct area. These results suggest that lomitapide treatment can significantly improve nerve injury after cerebral ischemia and promote the recovery of nerve function.

### 3.3 Lomitapide activates autophagy in neurons in ischemic penumbra of cortices

Autophagy plays a neuroprotective role in ischemic stroke by promoting clearance of damaged proteins and organelles, conducive to energy recycling and cell defense.^15^ Whether autophagy can be completed normally is the basis of a variety of physiological and pathological processes in the body and is also closely associated with the pathogenesis and prognosis of many diseases. Many studies have shown that autophagy is involved in the occurrence and development of ischemic stroke, and its regulatory mechanism involves complex signal transduction pathways.^15^, ^16^, ^17^ After cerebral ischemia–reperfusion injury, oxidative stress, inflammation, and other factors are important in inducing autophagy, whereas ischemia time, ischemia–reperfusion stage, and other factors are directly related to the role of autophagy in cerebral ischemia–reperfusion injury.^15^ Therefore, autophagy regulation may be a new strategy for stroke treatment. Autophagy plays a dual role in cerebral ischemia, which is related to the different animal models and drugs used for induction. In studying autophagy and cerebral ischemia injury, autophagy caused by different drugs may be enhanced or blocked; however, these drugs may have protective effects, and the specific mechanism of action needs to be elucidated.

**FIGURE 3:**
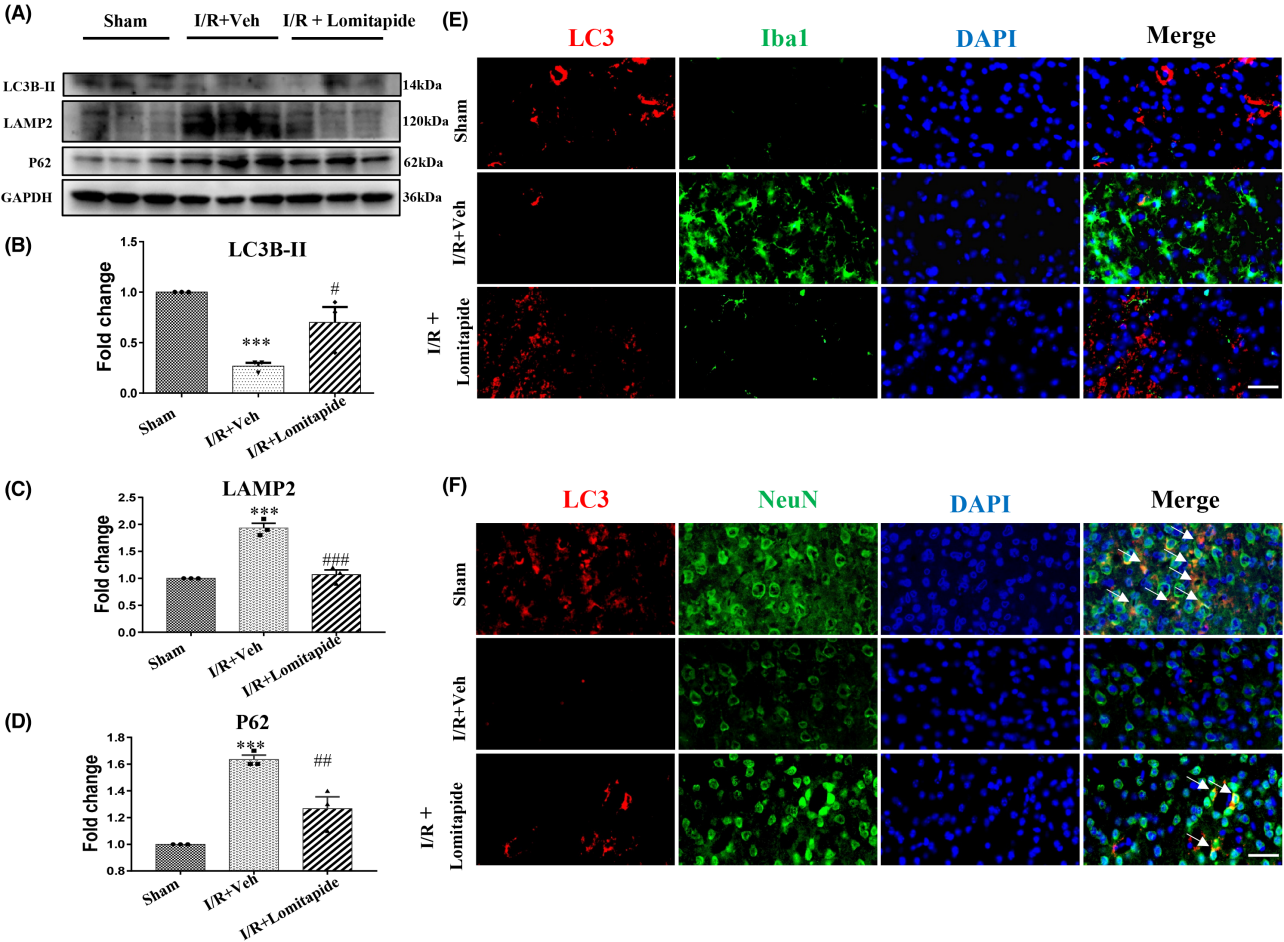
Lomitapide promotes autophagy activation in neurons in ischemic penumbra of cortices. Western blot detection (A) and quantitative analysis of LC3 (B), LAMP2 (C), and P62 (D) at 14 days after MCAO. *n* = 3, *p ≤ 0.05, ***p ≤ 0.001 vs. sham group; ^#^ *p* ≤ 0.05, ^##^ *p* ≤ 0.01, ^###^ *p* ≤ 0.001 vs. I/R + Veh group, one‐way ANOVA followed by Tukey's multiple comparison test. (E) Representative images of Iba1‐stained microglia (green) and LC3‐positive cells (red) and (F) representative immunofluorescence images showing co‐localization of NeuN‐stained neurons (green) and LC3‐positive cells (red) in the peri‐infarction areas from I/R mice in different groups 14 days after MCAO. DAPI (blue) indicates cell nuclei. The white arrows point to the co‐labeling cells. Scale bar, 100 μm

In this study, Western blotting revealed a significant increase in the expression of LAMP2 (*p* = 0.0002) and P62 (*p* = 0.0004) in the I/R group compared with sham group, while LC3 expression (*p* = 0.0005) was significantly decreased in the mouse brain at 14 days after infarction, indicating that ischemia and hypoxia inhibited autophagy activity of neurons. However, compared with the I/R group, the expression of LC3 (*p* = 0.0482) in the I/R + Lomitapide group was significantly increased, while expression of LAMP2 (*p* = 0.0004) and P62 (*p* = 0.0074) was decreased, as shown in Figure 3A–D, suggesting that lomitapide can increase autophagy activity of neurons after cerebral infarction. Moreover, immunofluorescence analysis showed that LC3 was mainly co‐localized with NeuN, but not with Iba1, indicating high autophagy activity of neurons in the brain tissue. After I/R, autophagy activity of neurons was predominantly inhibited, whereas lomitapide could promote autophagy activity of neurons (Figure 3E,F). This mechanism could be the potential mode of action of lomitapide to reduce cerebral ischemia injury and improve neural function.

### 3.4 Lomitapide promotes autophagic flux and inhibits apoptosis of neuronal cells during N2a‐BV2 co‐cultivation

**FIGURE 4:**
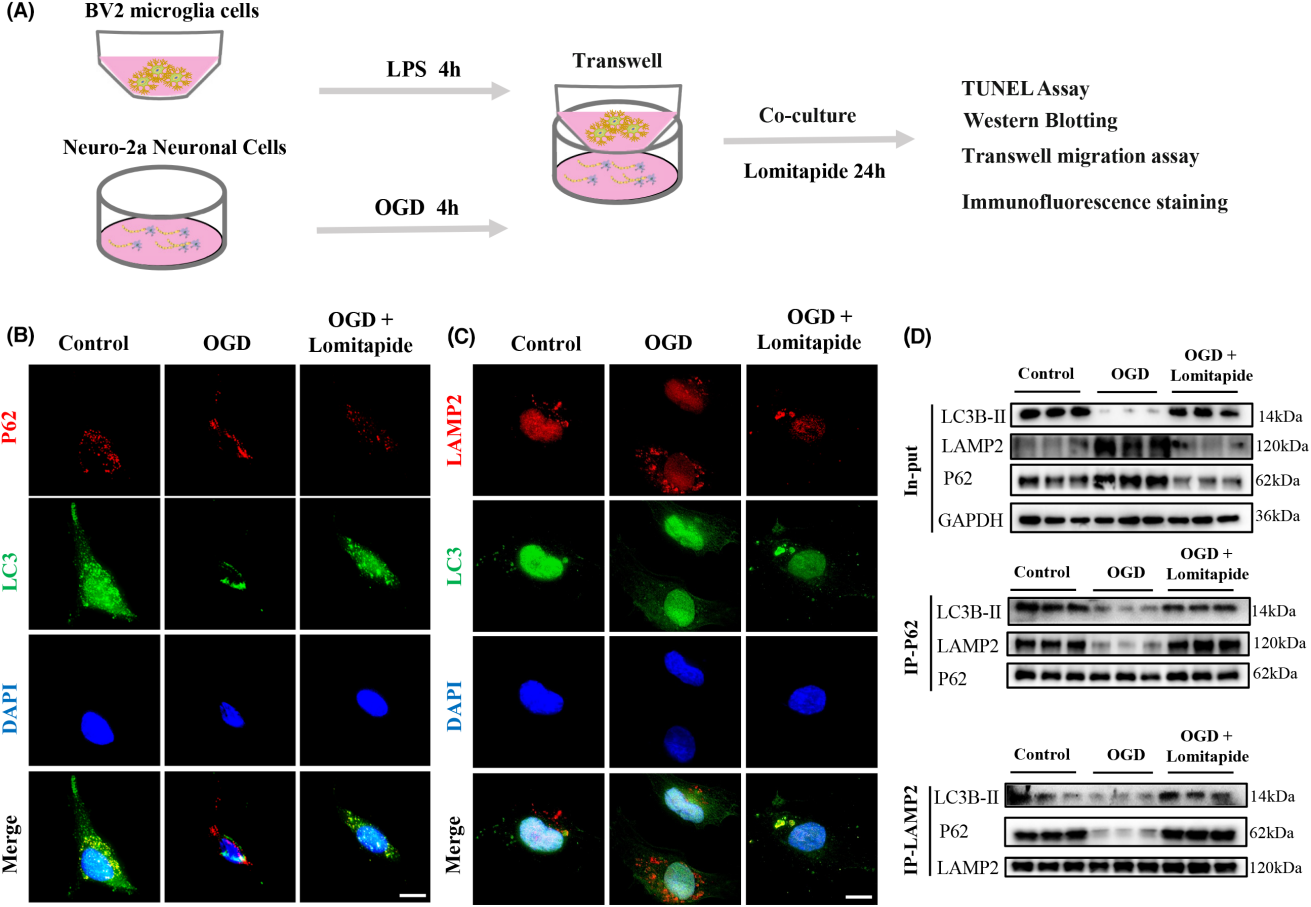
Lomitapide promotes autophagy flux of neurons in N2a‐BV2 co‐cultivation. (A) A representative flowchart of the experimental procedure in the N2a‐BV2 co‐cultivation. Representative cell immunofluorescence images showing the subcellular co‐localization of (B) P62 (red)‐LC3B (green) and (C) LAMP2 (red)‐LC3B (green). DAPI (blue) indicates cell nuclei. The bars indicate 15 μm. (D) Co‐immunoprecipitation with anti‐P62 and anti‐LAMP2 antibodies show the interaction between the autophagy proteins (P62, LC3B, and LAMP2)

**FIGURE 5:**
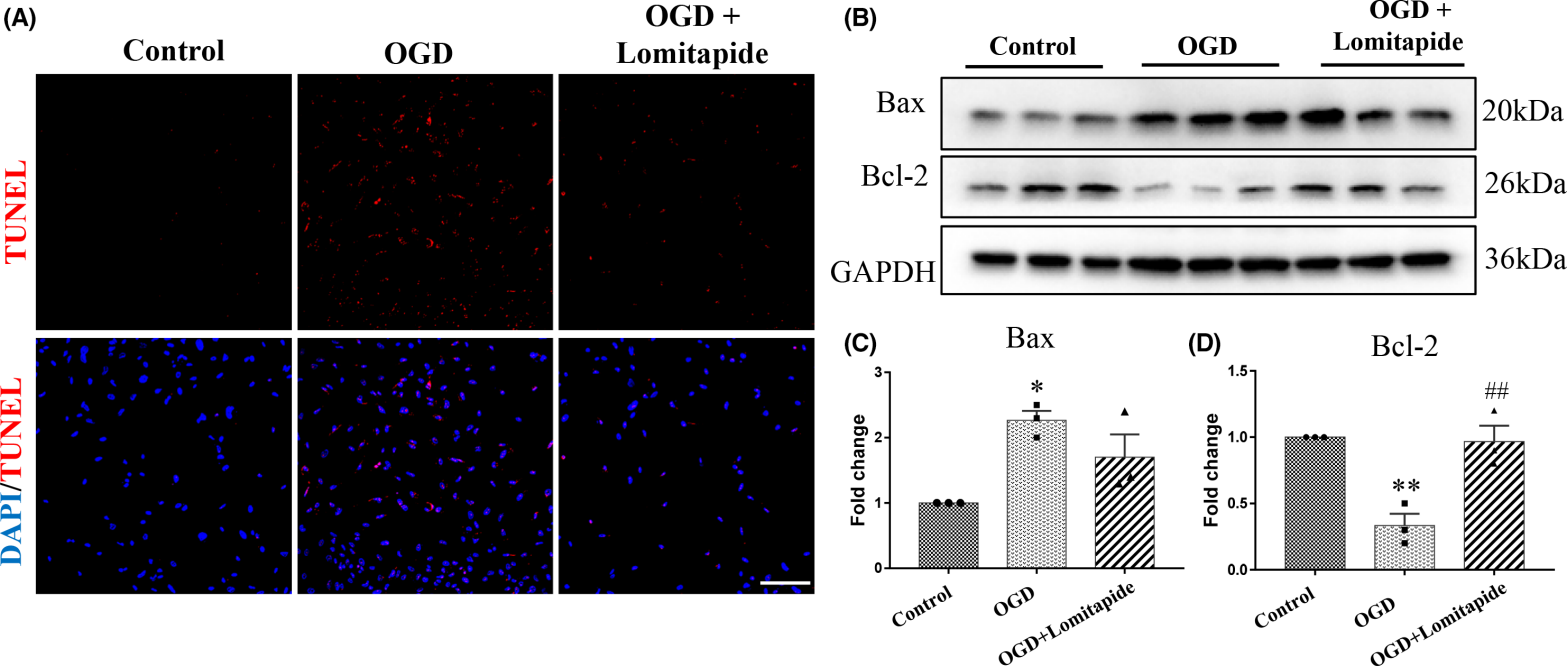
Lomitapide inhibits apoptosis of N2a neurons in N2a‐BV2 co‐cultivation. Representative images of TUNEL staining in Neuro‐2a cells (A). Western blot detection (B) and quantitative analysis of the apoptosis‐associated proteins Bax (C) and Bcl‐2 (D). *n* = 3, *p ≤ 0.05, **p ≤ 0.01, ***p ≤ 0.001 vs. Control group; ^##^ *p* ≤ 0.01, ^###^ *p* ≤ 0.001 vs. OGD group, respectively, one‐way ANOVA followed by Tukey's multiple comparison test. The bars indicate 100 μm

LPS‐induced microglia were co‐cultured with OGD‐treated neurons in vitro to simulate cerebral ischemia model and verify the changes in autophagy‐related protein expression (Figure 4A). We found that in the OGD group, LC3 did not co‐locate with P62 and LAMP2, while in the OGD + Lomitapide group, LC3 co‐located with P62 and LAMP2 (Figure 4B,C). Co‐immunoprecipitation experiments (Figure 4D) showed that expression of P62 and LAMP2, but not LC3, in the OGD group was higher than that in the control group, indicating blockade of autophagic flux. Lomitapide treatment reduced LAMP2 and P62 expression but increased LC3 expression, reflecting lomitapide‐related stimulation of autophagy and lysosomal degradation activity. These results indicate that lomitapide could alleviate blockage of autophagic flux caused by OGD in neurons. In addition, TUNEL assay was used to detect apoptosis of neuronal cells, and lomitapide was found to alleviate apoptosis induced by OGD (Figure 5A). We determined the expression of the apoptosis‐associated proteins Bax and Bcl‐2 and found that lomitapide significantly increased the expression of Bcl‐2 (*p* = 0.0048) compared with the OGD treatment group (Figure 5B–D). This finding suggests that the protective effect of lomitapide may be related to the promotion of autophagy flow and inhibition of apoptosis.

### 3.5 Lomitapide decreased the migration of microglia in vivo and vitro

**FIGURE 6:**
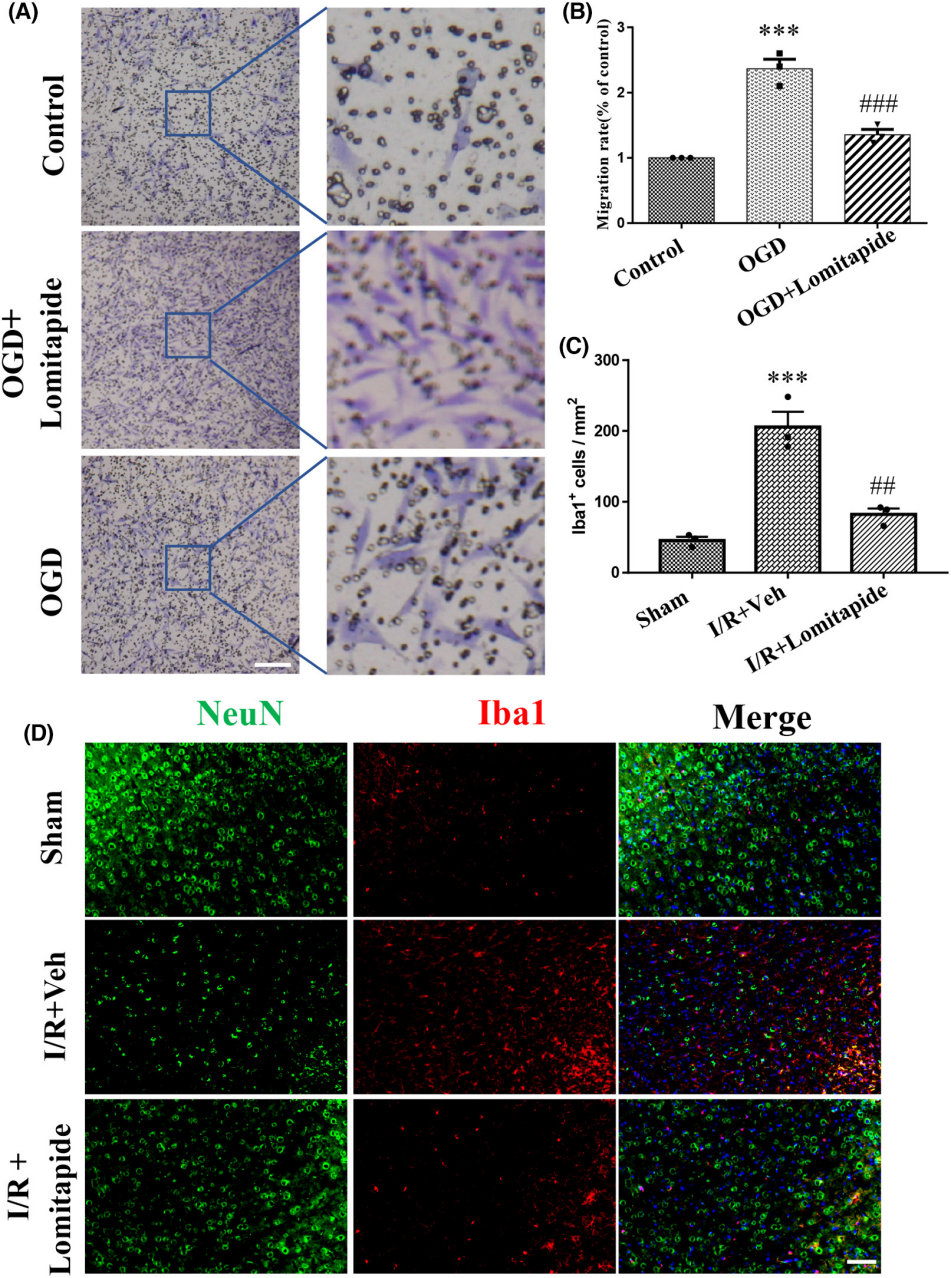
Lomitapide inhibits migration of microglia/macrophage in vivo and vitro. The inhibitory effect of lomitapide on BV2 microglia migration was recorded using (A) a camera and was measured using (B) a microplate reader. (C) Quantification of Iba1‐postive cells around the ischemic core area. Data are expressed as means ± SEM. *n* = 3/each group. ***p < 0.001 vs. Sham group, ^##^ *p* ≤ 0.01, vs. I/R + Veh group. (D) Representative immunofluorescence images of co‐localization of Iba1 (red) and NeuN (green) in the sham, I/R + Veh, and I/R + Lomitapide groups. DAPI (blue) indicates cell nuclei. The bars indicate 50 μm

Under local inflammatory stimulation, microglia not only change their phenotype but also their migration ability, enabling them to migrate and infiltrate inflammatory lesions.^18^ When ischemic brain injury occurs, microglia/macrophage activated by ischemic stimulation can migrate to the injured areas of the brain. Several studies have shown that LPS can induce microglia toward pro‐inflammatory M1 polarization.^19^, ^20^, ^21^, ^22^ We used LPS to induce microglia and co‐cultured them with OGD‐treated neurons. OGD treatment induced the migration of microglia, while lomitapide treatment (vs. OGD group, *p* = 0.0007) significantly reduced the migration of microglia to neurons (Figure 6A,B). This result was verified in vivo through immunofluorescence. Compared with sham group, the number of microglia/macrophage in the peripheral area of cerebral infarction in I/R group was significantly increased, while after treatment with lometatide, the over aggregation of microglia/macrophage in the peripheral area of cerebral infarction in I/R group was significantly reduced. (Figure 6C,D). These results suggest that the protective effect of lomitapide on cerebral ischemia may be related to the inhibition of migration of pro‐inflammatory microglia.

## 4 DISCUSSION

Drugs approved for marketing are often of low or non‐toxic nature, as supported by biological and clinical data. Therefore, discovering new applications of approved drugs can greatly shorten the development cycle and reduce the cost and risk of development. Lomitapide mesylate is a listed drug, recognized as safe, and non‐toxic or has low toxicity to human body. In the current study, we found that lomitapide has a protective effect on neuronal cells induced by ischemia and hypoxia. Moreover, lomitapide can improve the damage to sensory motor ability caused by ischemia and hypoxia in the MCAO mouse model, while promoting the coordination and integration of movement in mice. Continuous treatment with lomitapide for 14 days after MCAO reduced the degree of brain injury and significantly improved the survival rate of mice. These results suggest that lomitapide, a lipid‐lowering drug, can be repositioned as a potential therapeutic drug for stroke treatment.

Ischemic stroke is caused by ischemia and hypoxia of brain tissue around the vascular supply area, due to interruption of local blood supply. It can cause a series of serious pathophysiological changes, including irreversible damage of neurons, and self‐repair of neurons. In addition to apoptosis and necrosis, autophagy is also involved, although its specific mechanism is not completely clear. Autophagy often plays the role of a double‐edged sword in cells. Appropriate autophagy is considered to have a protective effect on ischemic neurons, whereas excessive autophagy is considered to promote cell death.^15^ Acute cerebral ischemia can lead to a reduction in oxygen and glucose supply, resulting in a series of metabolic changes, including neuronal death caused by insufficient cell energy supply.^23^, ^24^ Autophagy is also involved in the neuronal death induced by ischemia and hypoxia. Some studies indicate that inducing autophagy in early ischemia can protect neurons and reduce ischemia–reperfusion injury^25^, ^26^, ^27^, ^28^; others suggest that the inhibition of autophagy can effectively protect neurons from hypoxia ischemia‐induced damage.^29^, ^30^ These contrasting results are not uncommon and may be attributed to the animal or cell model used as well as the choice of drugs. In addition, early studies confused autophagy with autophagic flux block. In this study, we detected three autophagy‐related proteins, LC3, P62, and LAMP2, and monitored changes in autophagic flux. We found that 14 days after MCAO, the autophagic flux of neuronal cells was inhibited. Lomitapide treatment for 14 days increased the expression of LC3, reduced the expression of P62 and LAMP2, and promoted autophagic flux. Additionally, lomitapide promoted co‐localization of the autophagy‐associated proteins P62/LC3/LAMP2 in the ischemic hypoxic cell model, and this result was further verified by its interaction in co‐IP assay. In the co‐IP experiment, we found that lomitapide could promote the interaction between LC3‐II and LAMP2/p62 but did not detect the expression of LC3‐I. We also found that lomitapide inhibited apoptosis induced by OGD. This finding shows that lomitapide may play a neuroprotective role by promoting neuronal autophagic flux. Therefore, studying the regulatory mechanism of lomitapide in autophagy after ischemic stroke and inhibiting cell death caused by cerebral ischemia by regulating autophagy may become an effective auxiliary method for the treatment of patients with hemorrhagic stroke.

Neuroprotection is a complex process that involves multiple targets, and although the mechanisms of action are different, they are interrelated. So far, there is no specific treatment for ischemic stroke using neuroprotection‐related molecular targets. Innate microglia constitute the first immune defense line in the brain. After acute cerebral ischemia, microglia/macrophage can be rapidly activated and polarized into two different phenotypes, namely pro‐inflammatory M1 type (classical activation type) and antiinflammatory M2 type (alternative activation type), playing the dual role of tissue damage and neuroprotection. Therefore, regulating the different phenotypes and functions of microglia/macrophage is an important target for alleviating ischemic brain injury and improving the prognosis of stroke, which is also a research hotspot in the field of stroke in recent years.^31^, ^32^ Stimulated by local inflammation, microglia/macrophage can not only change their phenotype but also change their migration ability. M1 type microglia/macrophage migrate and infiltrate into inflammatory lesions, release cytokines, destroy neuronal cells, and aggravate brain tissue damage.^33^, ^34^ Therefore, further studies on the molecular mechanism of regulation of migration and polarization of microglia/macrophage may provide a new strategy for the treatment of ischemic stroke.^35^, ^36^ Several studies have found that LPS can induce transformation of microglia into M1.^19^, ^22^ In the current study, LPS‐induced microglia were co‐cultured with OGD‐induced neuronal cells, which enhanced the migratory ability of microglia toward neuronal cells. However, lomitapide treatment significantly inhibited the migration of microglia. We verified this result in vivo and found that lomitapide treatment could reduce microglial/macrophage overaggregation in the peripheral area of cerebral infarction in the I/R group mice. This finding shows that lomitapide can offer neuroprotection after ischemic brain injury by regulating the migration of microglia and provides evidence and ideas for the clinical application of lomitapide in the treatment of stroke.

## 5 LIMITATIONS AND FUTURE PERSPECTIVES

The sex differences in stroke have been paid growing attention. Studies have found that there are sex differences in stroke outcomes,^37^ epidemiology,^38^ stroke therapies,^39^ post‐stroke remodeling and functional recovery,^40^ and the mechanism affecting the sexual difference of stroke involves multiple levels of pathophysiology.^41^, ^42^, ^43^ An important limitation of our study is to use male mice only to study the protective mechanism of lomitapide. Sex differences still need to be considered in the future, which is of great significance to study the protective mechanism of lomitapide against ischemic brain injury. The period and level of autophagy determine the final fate of cells. A certain period and level of autophagy may offer advantages and avoid disadvantages for the cells, but serious autophagy can induce programmed cell death.^44^ At present, the molecular mechanism of its role transformation has not been clarified. Another limitation of our research is that we have not determined the changes in autophagy at different time points, and thus, further research in this direction is warranted. In the process of autophagy, LC3‐I covalentates with phosphatidylethanolamine under the action of ATG7 and ATG12‐ATG5‐ATG16L to form LC3‐II, which binds to the autophagosome membrane. Generally, the ratio of LC3‐II/LC3‐I is used as an indicator of autophagy activity.^45^ Therefore, the inability to detect LC3‐I expression in co‐IP experiments is also a limitation of this study.

## 6 CONCLUSION

In conclusion, this study shows that lomitapide has a therapeutic effect on ischemic nerve injury. It has an obvious protective effect on neuronal cells, mainly through two aspects: First, lomitapide promotes autophagic flux and reduces apoptosis of neuronal cells; second, lomitapide inhibits the migration of microglia/macrophage and then reduces further damage of neuronal cells. To our knowledge, this is the first study on the protective effect of lomitapide on ischemic nerve injury and can provide a new means of intervention and a theoretical basis for the treatment of cerebral ischemia–reperfusion injury.

## AUTHOR CONTRIBUTIONS

Yumin Luo designed the experiments. Yangmin Zheng and Yue Hu wrote the manuscript. Ziping Han, Feng Yan Rongliang Wang and Sijia Zhang performed the experiments and analyzed the data. All authors contributed to manuscript revision, reading, and approval of the submitted version.

## CONFLICT OF INTEREST

Dr Luo is an Editorial Board member of CNS Neuroscience and Therapeutics and a co‐author of this article. To minimize bias, they were excluded from all editorial decision making related to the acceptance of this article for publication.

## Supporting information


Appendix S1

## Data Availability

All processed data used in this study can be obtained from the cor‐responding author on reasonable request. The original, uncropped image of each cropped gel/blot of the western blotting experiment in the study are shown in Appendix S1.
