# Supplementary material for: Lomitapide ameliorates middle cerebral artery occlusion‐induced cerebral ischemia/reperfusion injury by promoting neuronal autophagy and inhibiting microglial migration
Source: CNS Neurosci Ther. 2022 Sep 2;28(12):2183–94. doi: 10.1111/cns.13961 (PMC9627359; doi:10.1111/cns.13961)
Supplement: Supplementary file 1 — Appendix S1 [file CNS-28-2183-s001.pdf]

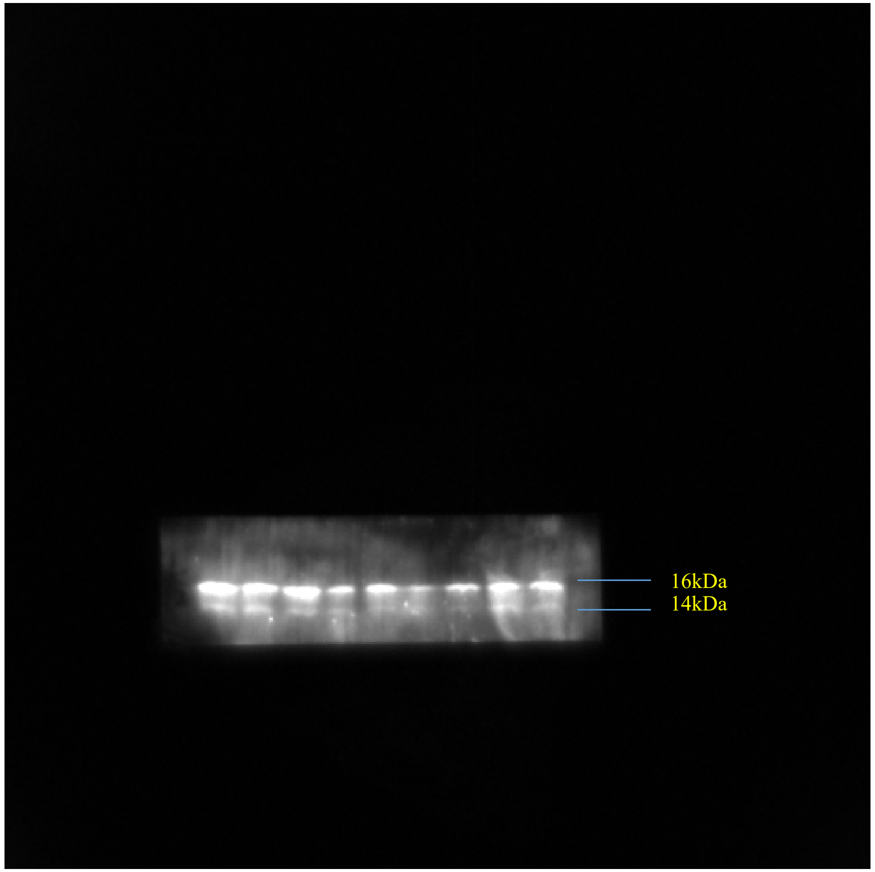

Full unedited gel/blot for Figure 2A (LC3)

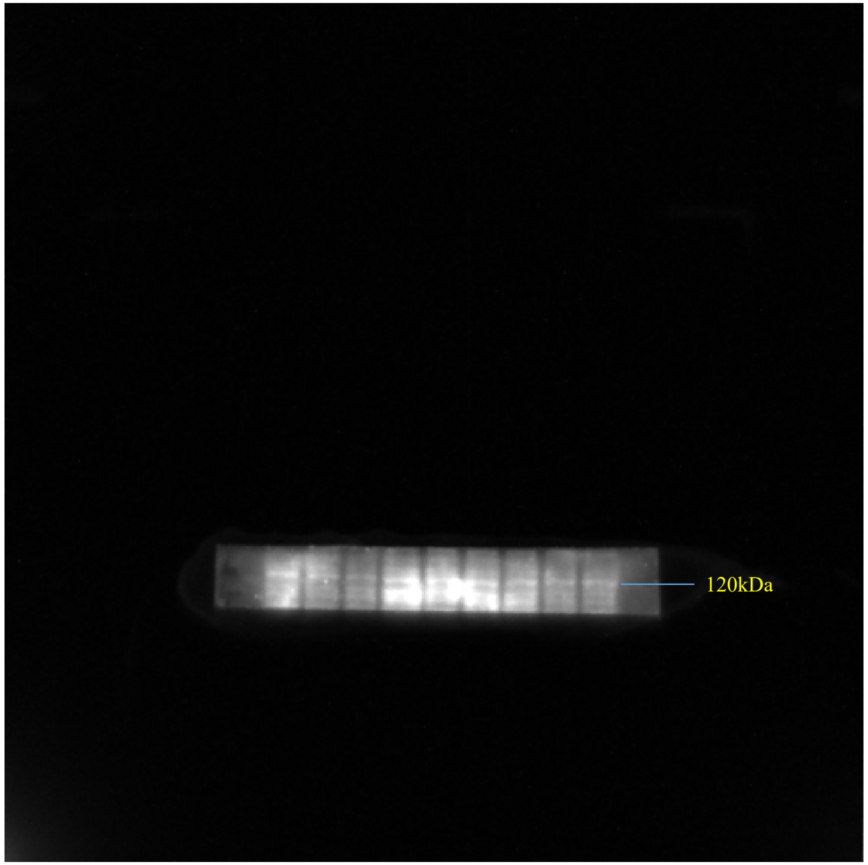

Full unedited gel/blot for Figure 2A (LAMP2)

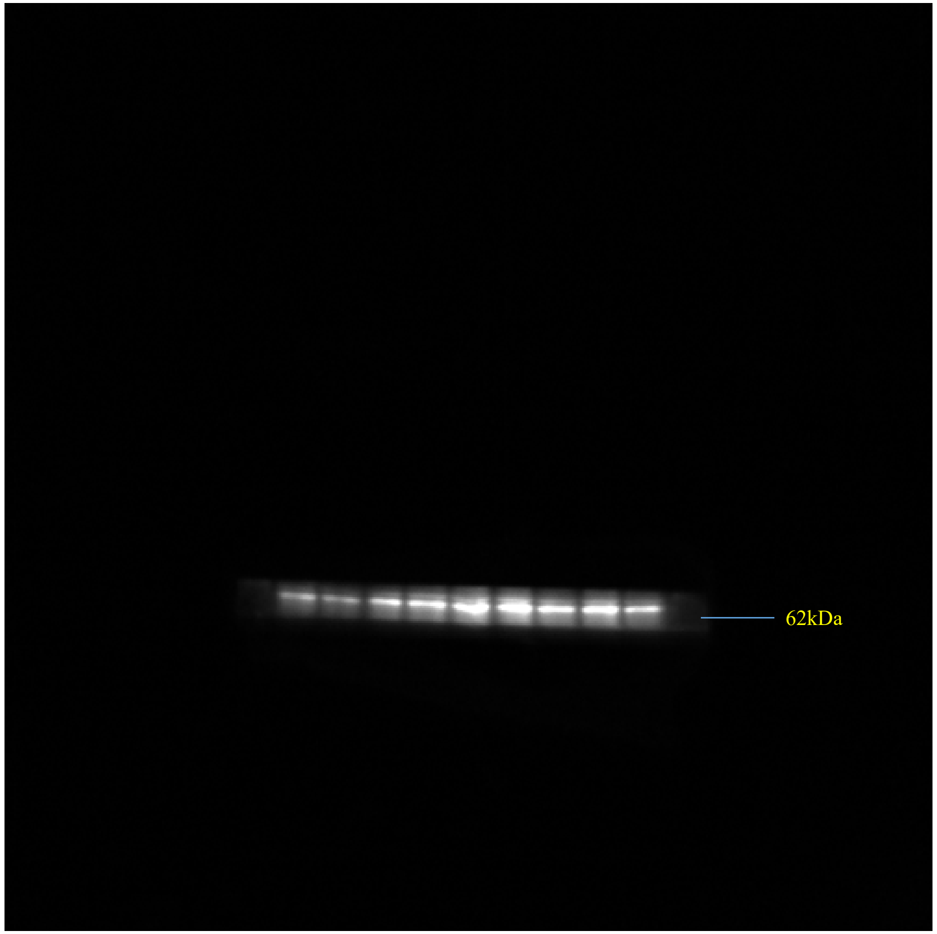

Full unedited gel/blot for Figure 2A (P62)

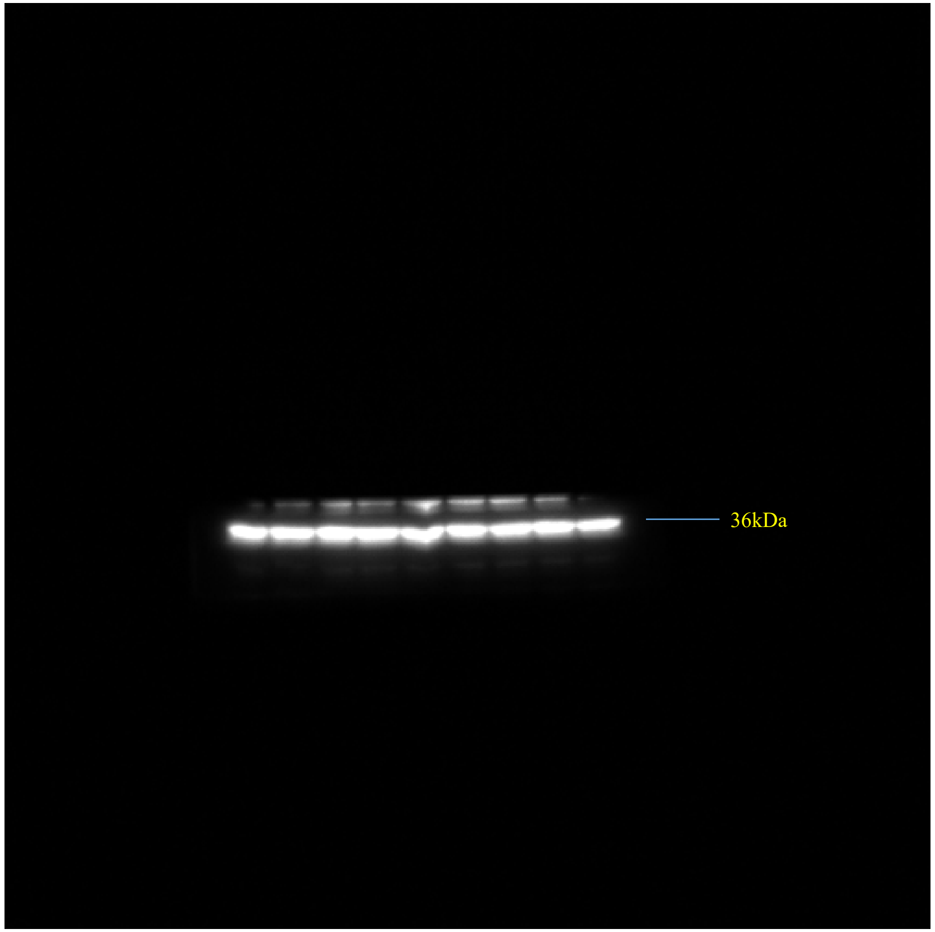

Full unedited gel/blot for Figure 2A (GAPDH)

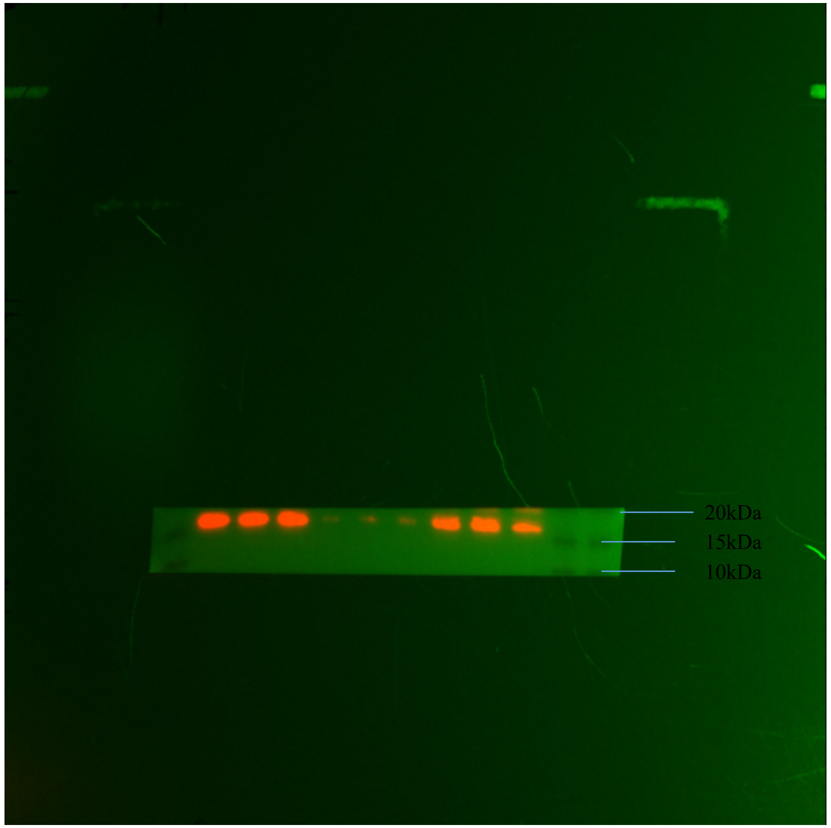

Full unedited gel/blot for Figure 4D In-put组 (LC3)

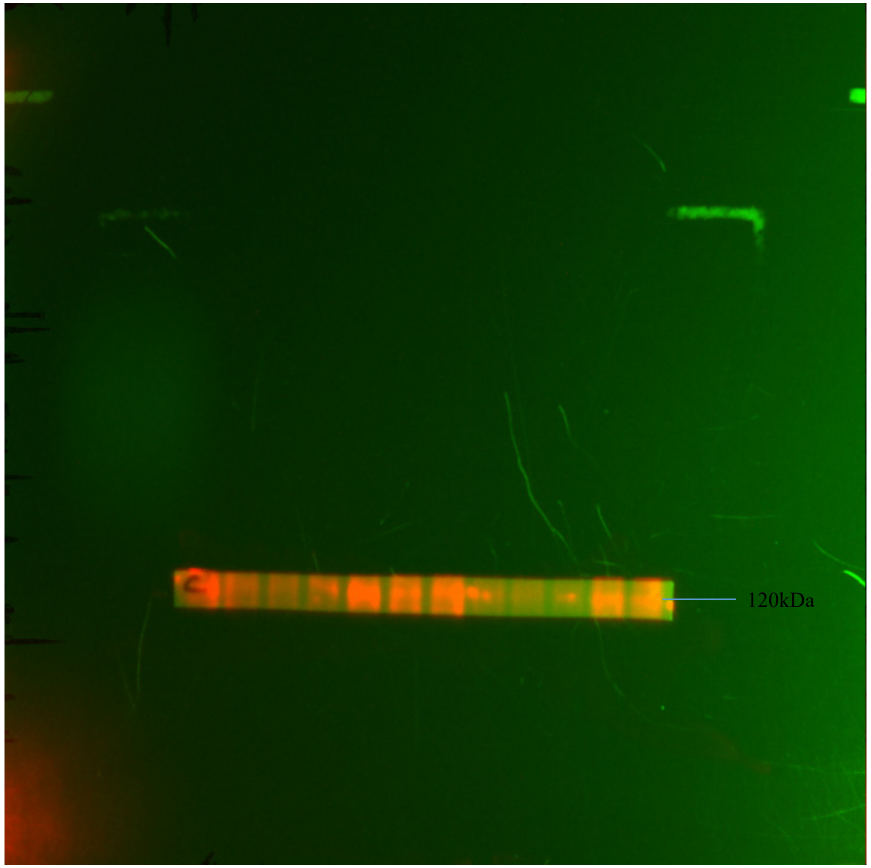

Full unedited gel/blot for Figure 4D In-put组(LAMP2)

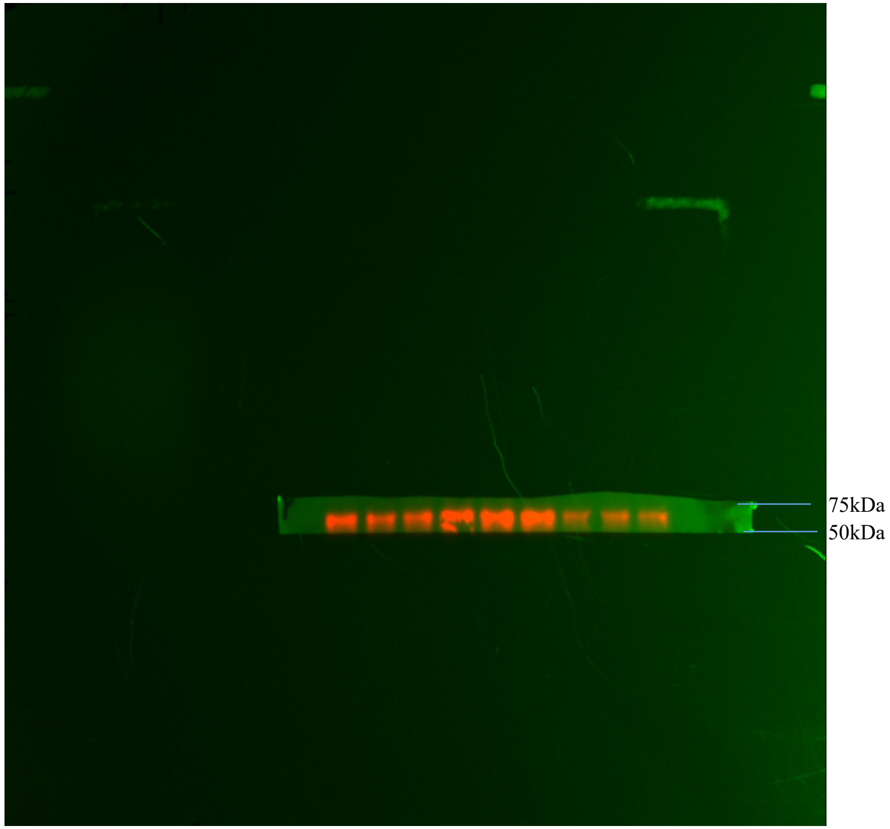

Full unedited gel/blot for Figure 4D In-put组 (P62)

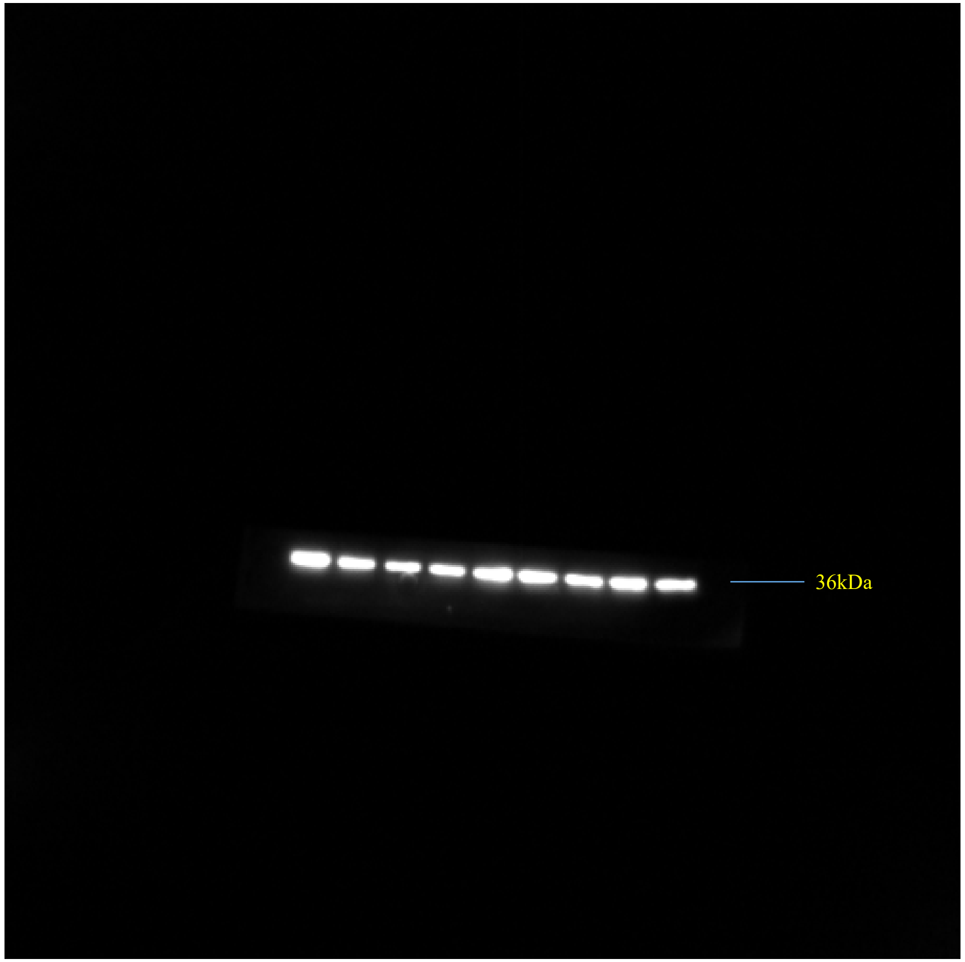

Full unedited gel/blot for Figure 4D In-put组 (GAPDH)

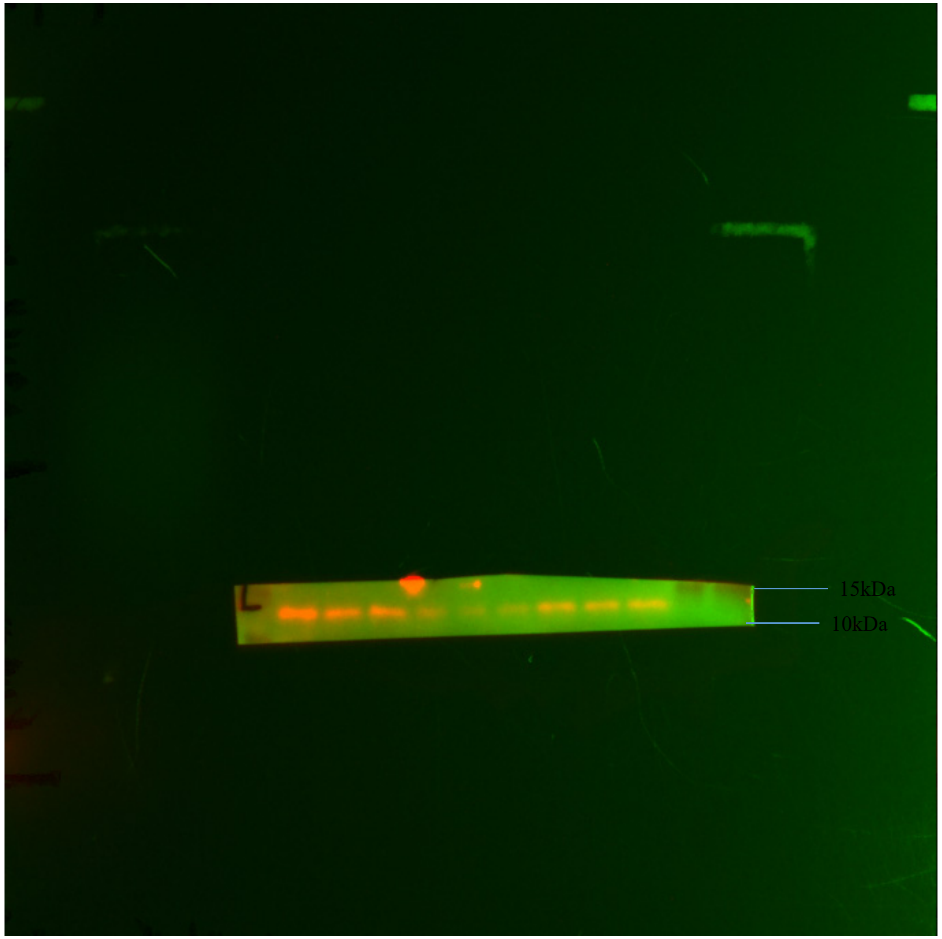

Full unedited gel/blot for Figure 4D Ip-p62组 (LC3)

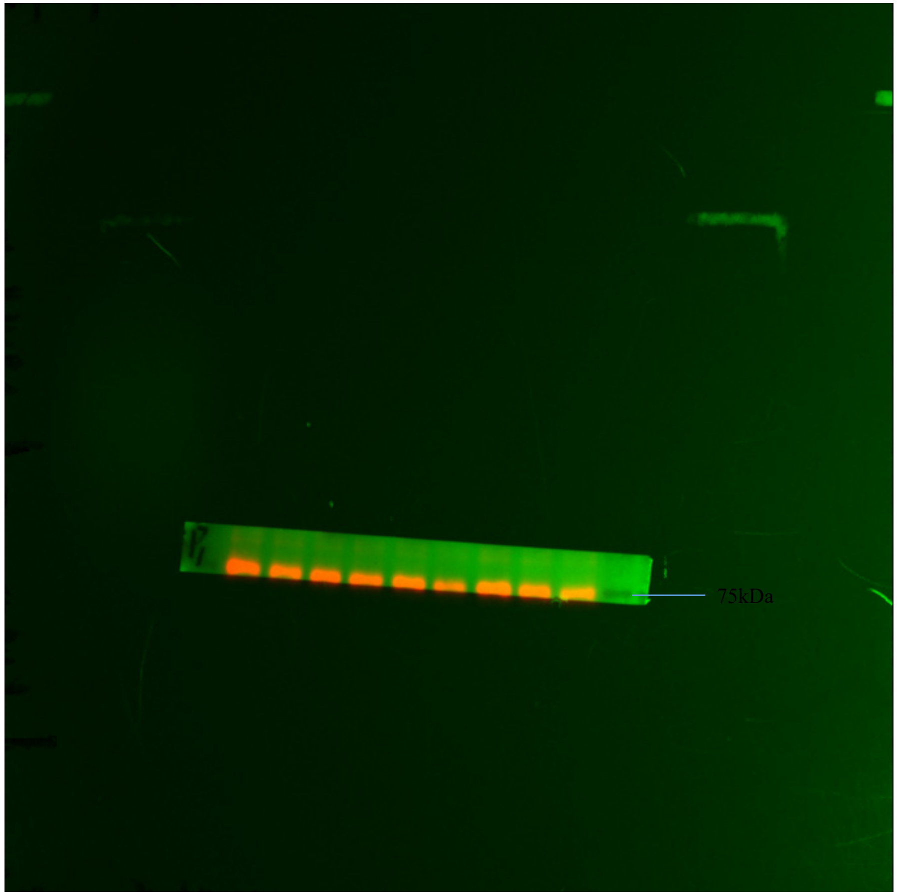

Full unedited gel/blot for Figure 4D Ip-p62组 (p62)

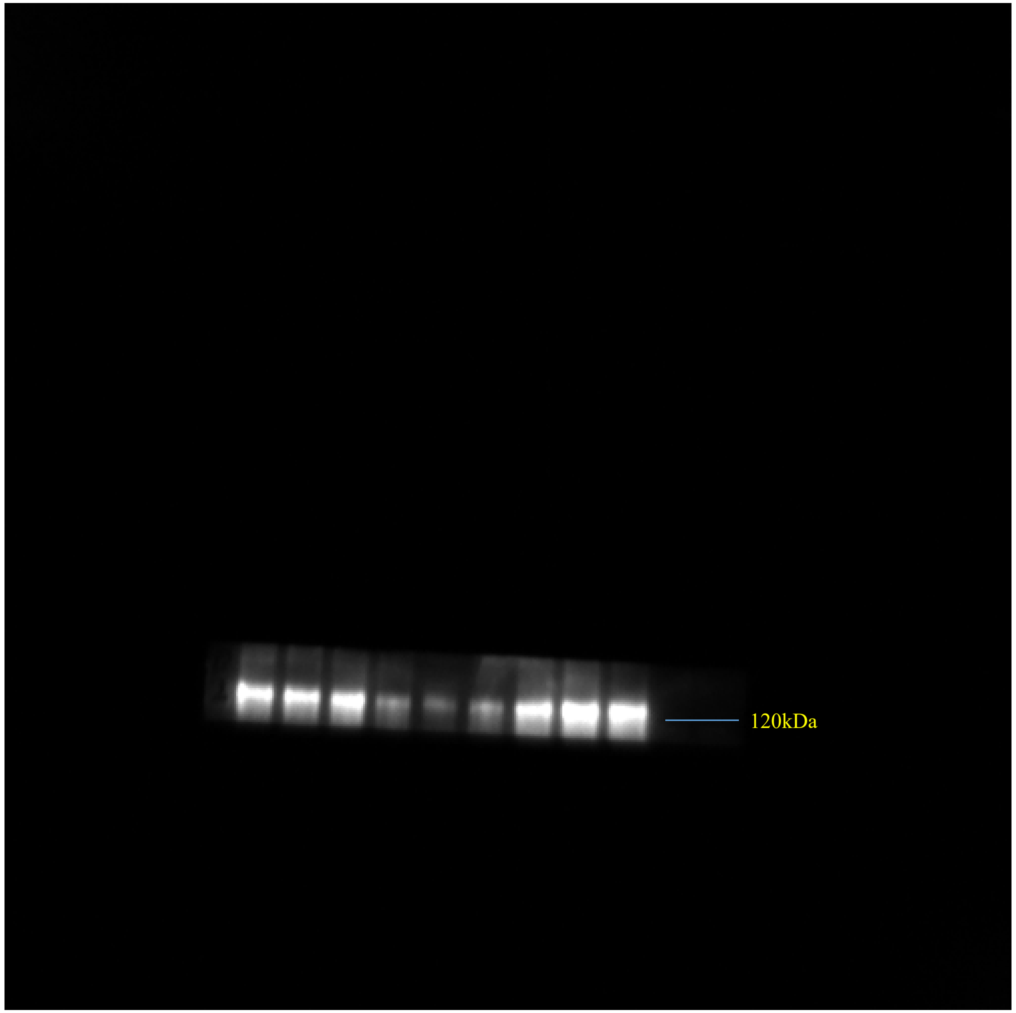

Full unedited gel/blot for Figure 4D Ip-p62组 (LAMP2)

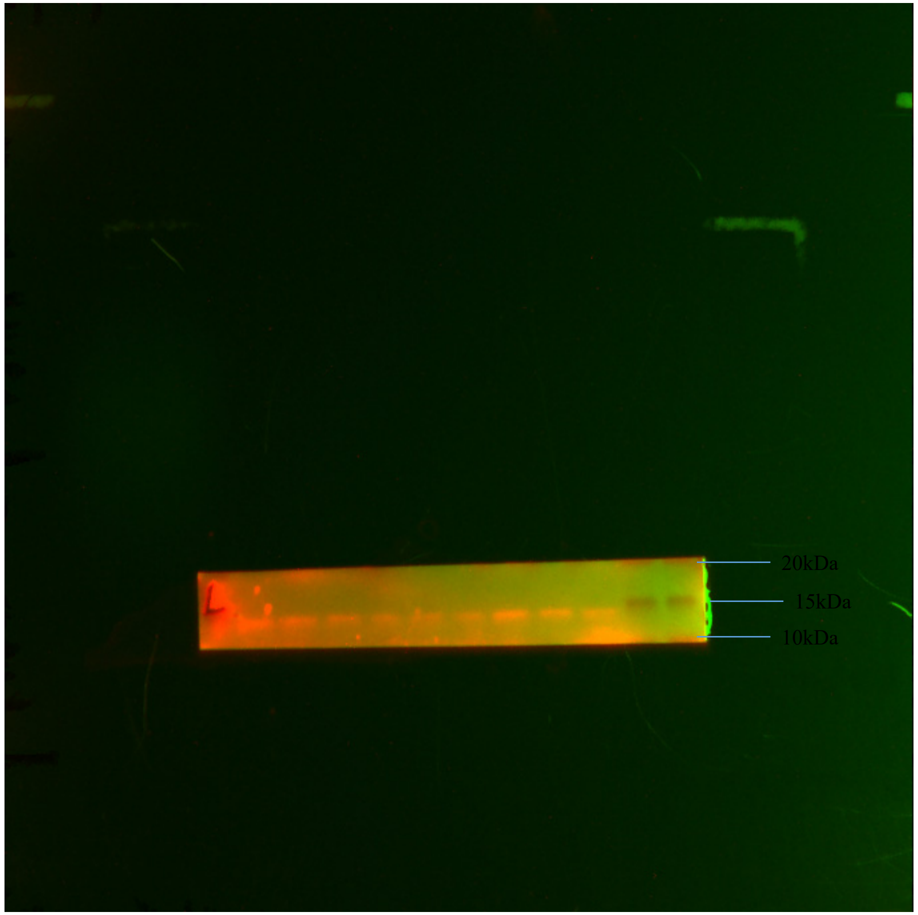

Full unedited gel/blot for Figure 4D Ip-LAMP2组 (LC3)

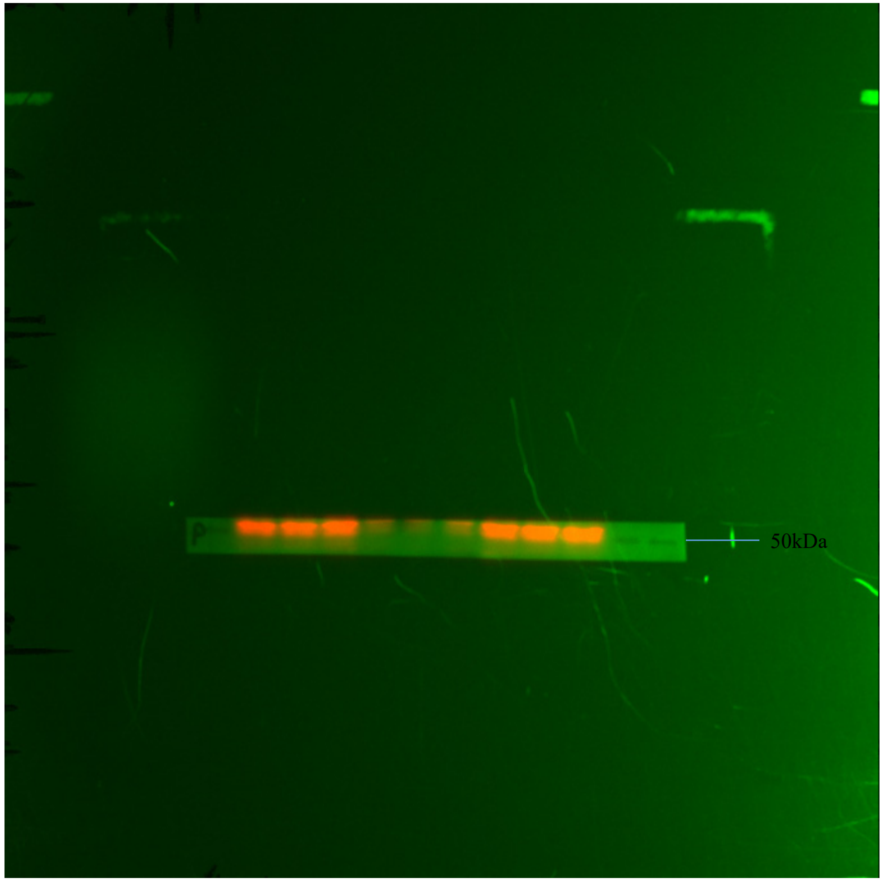

Full unedited gel/blot for Figure 4D Ip-LAMP2组 (p62)

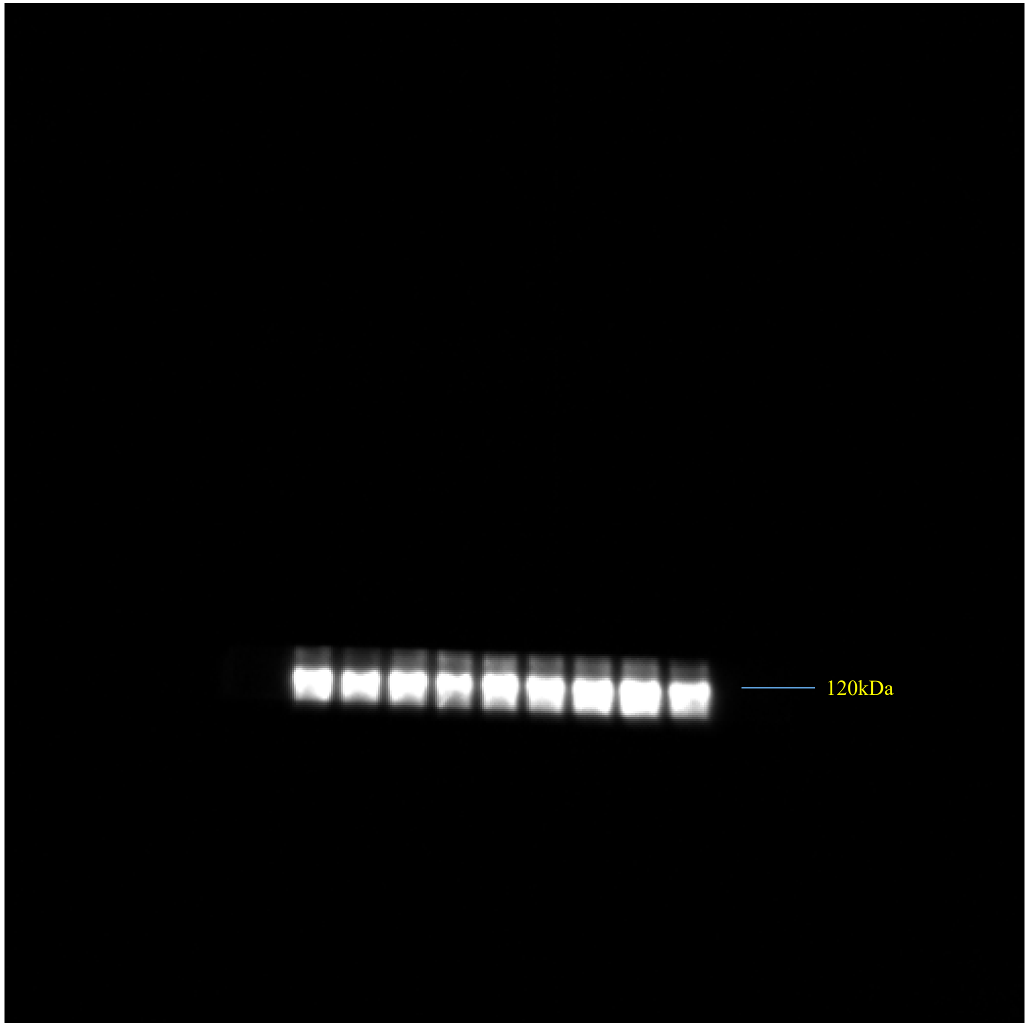

Full unedited gel/blot for Figure 4D Ip-LAMP2组 (LAMP2)

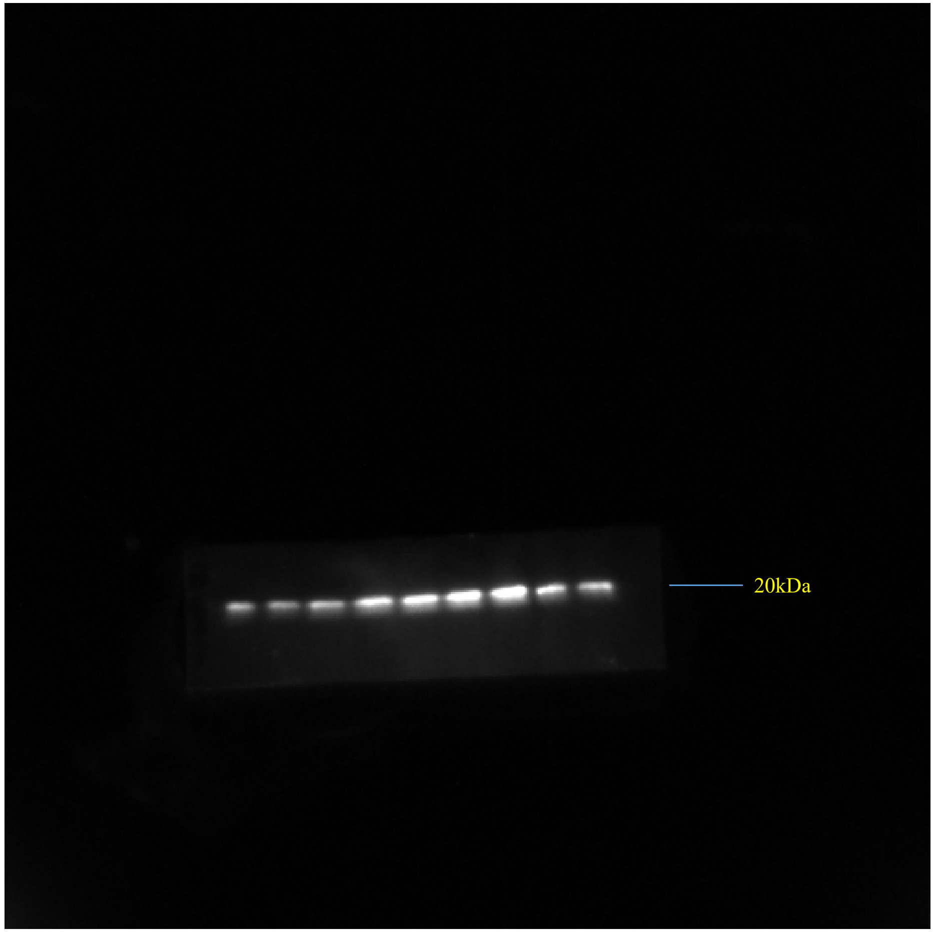

Full unedited gel/blot for Figure 5B (Bax)

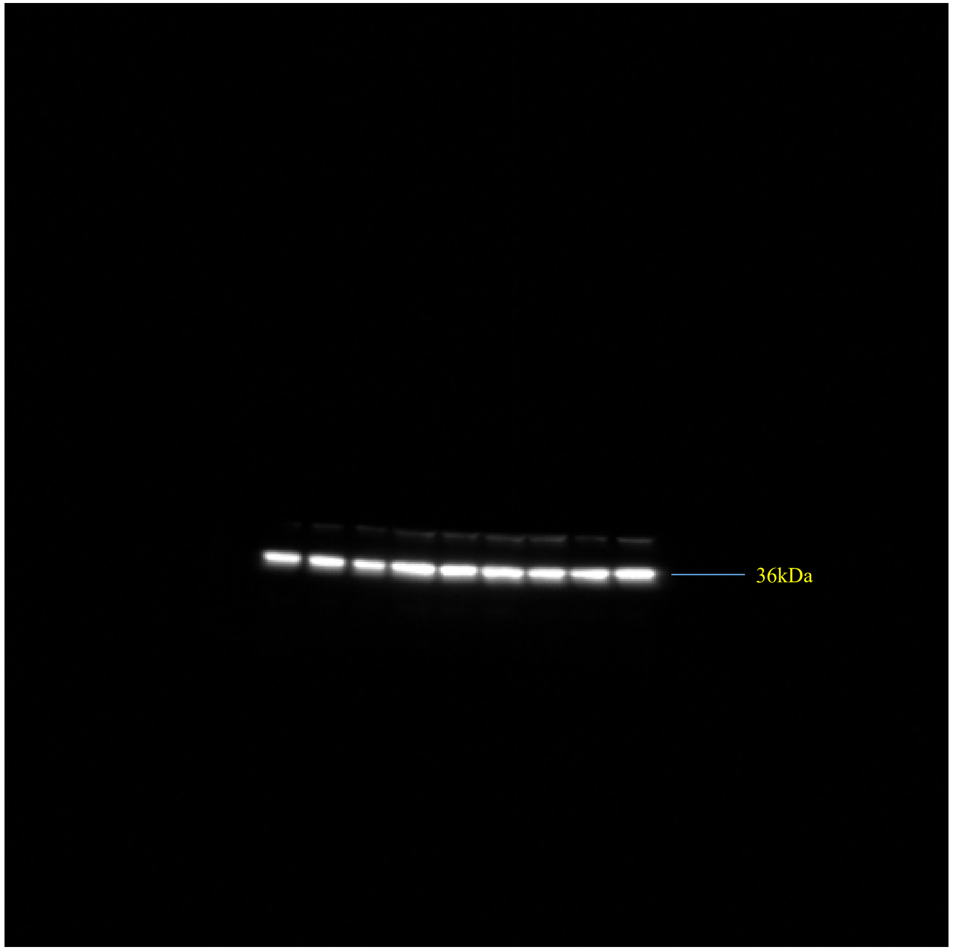

Full unedited gel/blot for Figure 5B (GAPDH)

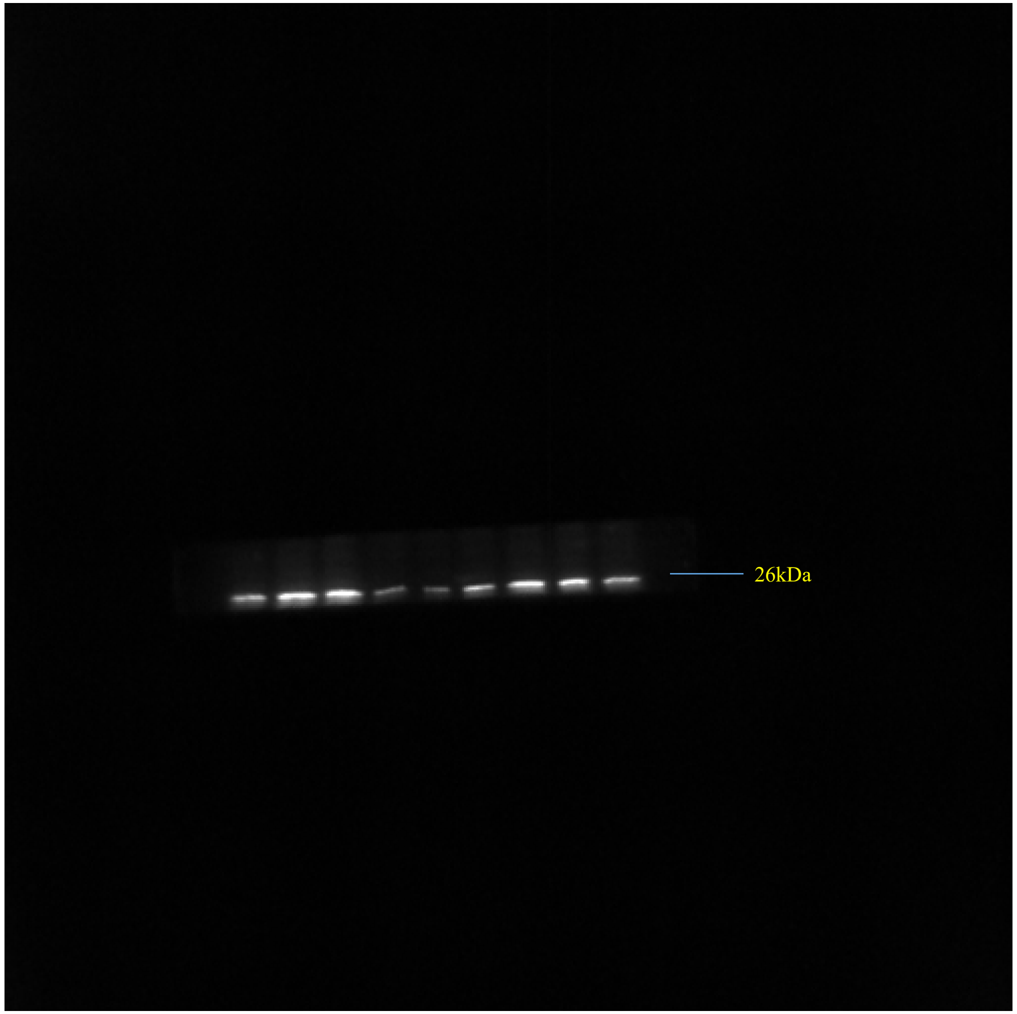

Full unedited gel/blot for Figure 5B (Bcl-2)
